# Macular Ganglion Cell -Inner Plexiform Layer Thickness Is Associated with Clinical Progression in Mild Cognitive Impairment and Alzheimers Disease

**DOI:** 10.1371/journal.pone.0162202

**Published:** 2016-09-06

**Authors:** Seong Hye Choi, Sang Jun Park, Na Rae Kim

**Affiliations:** 1 Department of Neurology, Inha University School of Medicine, Incheon, Korea; 2 Department of Ophthalmology and Inha Vision Science Laboratory, Inha University School of Medicine, Incheon, Korea; Universitatsklinikum Leipzig, GERMANY

## Abstract

**Purpose:**

We investigated the association of the macular ganglion cell-inner plexiform layer (GCIPL) and peripapillary retinal nerve fiber layer (RNFL) thicknesses with disease progression in mild cognitive impairment (MCI) and Alzheimer’s disease (AD).

**Methods:**

We recruited 42 patients with AD, 26 with MCI, and 66 normal elderly controls. The thicknesses of the RNFL and GCIPL were measured via spectral-domain optic coherent tomography in all participants at baseline. The patients with MCI or AD underwent clinical and neuropsychological tests at baseline and once every year thereafter for 2 years.

**Results:**

The Clinical Dementia Rating scale-Sum of Boxes (CDR-SB) score exhibited significant negative relationships with the average GCIPL thickness (*β* = -0.15, *p* < 0.05) and the GCIPL thickness in the superotemporal, superonasal, and inferonasal sectors. The composite memory score exhibited significant positive associations with the average GCIPL thickness and the GCIPL thickness in the superotemporal, inferonasal, and inferotemporal sectors. The temporal RNFL thickness, the average and minimum GCIPL thicknesses, and the GCIPL thickness in the inferonasal, inferior, and inferotemporal sectors at baseline were significantly reduced in MCI patients who were converted to AD compared to stable MCI patients. The change of CDR-SB from baseline to 2 years exhibited significant negative associations with the average (*β* = -0.150, *p* = 0.006) and minimum GCIPL thicknesses as well as GCIPL thickness in the superotemporal, superior, superonasal, and inferonasal sectors at baseline.

**Conclusions:**

Our data suggest that macular GCIPL thickness represents a promising biomarker for monitoring the progression of MCI and AD.

## Introduction

Alzheimer’s disease (AD) is the most common age-related dementia and is characterized by the accumulation of amyloid-β protein (Aβ) plaques as well as aggregates of hyperphosphorylated tau as neurofibrillary tangles in the brain.[1] The pathophysiological process of AD begins many years prior to detectable cognitive impairment.[2] The transitional phase is clinically recognized as preclinical AD and mild cognitive impairment (MCI).[2, 3] MCI has multiple etiologies and is categorized into amnestic and non-amnestic subtypes.[4] Amnestic MCI is considered a degenerative condition that may represent prodromal AD.[3] Recently, there has been a remarkable growth in AD biomarkers, including cerebrospinal fluid (CSF) measurement of a lower Aβ_42_ level, positron emission tomography (PET) amyloid imaging, and the assessment of medial temporal lobe atrophy via brain magnetic resonance imaging (MRI).[3, 5] Nevertheless, researchers continue to search for new, less invasive and more cost-effective biological markers of AD.[6]

Many patients with AD experience a loss of visual acuity, a phenomenon that has no known mechanism. One of the most plausible explanations involves the loss of retinal ganglion cells (RGCs).[7] The loss or RGCs is part of the normal aging process. In AD, though, the loss of retinal ganglion cells is significantly greater and is accompanied by M-cell loss.[8] Postmortem studies of AD, in addition to the well-known occurrence of neurodegeneration in the brain, have revealed a significant loss of RGCs as well as abnormal RGC dendritic morphology and size.[9, 10] The accumulation of Aβ has also been reported inside and around RGCs in postmortem AD retinal specimens.[9]

Several studies have searched for in vivo evidence of retinal involvement in AD pathophysiology using optical coherence tomography (OCT). The OCT technique used to measure the peripapillary retinal nerve fiber layer (RNFL) and macular thickness has proven useful for the detection of significant reductions in retinal thickness in patients with AD and in those with MCI.[11–16] The spatial resolution of spectral domain OCT (SD-OCT) allows for the measurement of the RGC layer, which contains the RGC somata, as well as the inner plexiform layer (IPL), which contains the RGC dendrites (GCIPL). A recent study reported that macular GCIPL is a more sensitive marker than RNFL thickness for the assessment of neurodegeneration pathology in MCI or AD.[17]

A reduction in the retinal thickness, as measured by OCT, may be a promising biomarker for monitoring the progression of MCI and AD. However, there is a lack of longitudinal studies investigating the association between retinal thickness and the progression of AD and MCI. We hypothesize that GCIPL thickness at the macula is more strongly associated than RNFL thickness with the progression of AD and MCI. In this study, we examined the association of macular GCIPL and RNFL thickness with cognitive impairment and disease progression via cross-sectional and longitudinal analyses of MCI and AD patients.

## Materials and Methods

### Participants

Forty-two patients with AD, 26 patients with amnestic MCI, and 66 elderly control subjects participated in this study. The patients with AD or MCI were recruited at the memory clinic of Inha University Hospital, Incheon, Korea, between March 2012 and February 2015. The subjects with AD met the criteria for dementia according to the Diagnostic and Statistical Manual of Mental Disorders, 4^th^ edition (DSM-IV)[18], as well as the criteria for probable AD established by the National Institute on Aging-Alzheimer’s Association workgroups.[19] The diagnosis of MCI was in accordance with Petersen et al.’s criteria[4], listed as follows: 1) subjective memory complaints corroborated by an informant; 2) objective memory decline, as defined by a delayed recall score on the Seoul Verbal Learning test (SVLT) less than 1.5 standard deviations (SD) below the age- and education-adjusted normative means[20]; 3) normal general cognitive function, as defined by Clinical Dementia Rating (CDR) scale of 0.5,[21] and Mini-Mental State Examination (MMSE) scores more than 1.5 SD below the age- and education-adjusted normative means[22]; 4) normal functional activities; and 5) lack of a dementia diagnosis.

The participants were subjected to extensive neuropsychological testing, including the SVLT of a 12-word list, the Short Form of the Korean version of the Boston Naming Test (K-BNT), the copy and recall test of Rey Osterrieth Complex Figure (ROCF), the digit span forward and backward test, animal fluency, the Controlled Oral Word Association test (COWAT), and the Color-Word Stroop test.[20] They were also administered a brain MRI and routine biochemical and serological tests that included a thyroid function test, tests of serum vitamin B12 and folate levels, and a Venereal Disease Research Laboratory test. Patients with AD or MCI underwent clinical and neuropsychological evaluations at baseline and once every year thereafter for 2 years.

Normal controls were recruited at the ophthalmology clinic of Inha University Hospital, Incheon, Korea, during the same period. They were at least 60 years old and did not have subjective memory complaints nor a history of any of the following 28 diseases, symptoms or operations suggestive of a decrease in cognitive function: stroke or transient ischemic attack; seizures; Parkinson’s disease; multiple sclerosis; cerebral palsy; Huntington’s disease; encephalitis; meningitis; brain surgery; surgery to clear arteries to the brain; diabetes mellitus requiring insulin; poorly controlled hypertension; cancer other than skin cancer diagnosed within the past three years; shortness of breath while sitting still; use of home oxygen; heart attack with changes in memory, walking, or solving problems lasting at least 24 hours afterward; kidney dialysis; liver disease; hospitalization for mental or emotional problems within the past five years; current use of medications for mental or emotional problems; alcohol consumption greater than three drinks each day; abuse of drugs within the past five years; treatment for alcohol abuse within the past five years; unconsciousness for more than one hour other than during surgery; overnight hospitalization due to a head injury; illness causing a permanent decrease in memory or other mental functions; trouble with vision that prevents reading ordinary print even with glasses on; and difficulty understanding conversations due to hearing, even if wearing a hearing aid.[23]

All participants underwent a full ophthalmic examination, including Goldmann applanation tonometry, measurements of best-corrected visual acuity, automated refraction, slit lamp examination of the anterior segment, dilated fundus examination, fundus photography (Canon, Tokyo, Japan), and Cirrus OCT (Macular cube mode and optic disc mode). All examinations were performed during a single visit. All the selected participants had a best corrected visual acuity of 20/40 or better, with a refractive error between +3.00 and 6.00 diopters, an intraocular pressure of 21 mm Hg or lower, an open angle on gonioscopy, normal fundus examination, and normal-appearing optic nerve heads. We excluded patients with a history of glaucoma and the presence of glaucomatous characteristics based on fundus photographs, which included the following symptoms: localized retinal nerve fiber layer defect or glaucomatous optic disc changes such as diffuse or localized rim thinning, disc hemorrhage, a notch in the rim, or a vertical cup-to-disc ratio exceeding than that of the other eye by more than 0.2. Patients exhibiting media opacity and pathological retinal features were also excluded.

This study was conducted in accordance with the Declaration of Helsinki and with good clinical reporting practices. The study protocol and informed consent forms were reviewed and approved by the Institutional Review Board at Inha University Hospital before the study began. Prior to participation in the study, the participants gave their written informed consent to participation.

### OCT measurements

A Cirrus High-Definition OCT (HD-OCT, software version 6.0) was used to acquire one Optic Disc Cube protocol and one Macular Cube protocol. The Optic Disc Cube protocol is designed to position the cube scan on the optic nerve head. This protocol generates a cube of data through a 6-mm square grid by acquiring a series of 200 horizontal scan lines, each composed of 200 A scans (40,000 points). The RNFL thickness at each pixel was measured, and an RNFL thickness map was generated. A calculation circle 3.46 mm in diameter and consisting of 256 A-scans was automatically positioned around the optic disc. The mean and sectoral (temporal, superior, nasal and inferior) RNFL thicknesses were also measured.

Macular cube scans of an area of 6 x 6 mm^2^ were centered on the fovea using the macular cube 512 x 128 or 200 x 200 scan protocol. The built-in software was used to produce retinal thickness maps, which then were averaged over nine retinal subfields in a 6 mm diameter circle centered at the true fovea location, as defined by the Early Treatment Diabetic Retinopathy Study (ETDRS). The ETDRS areas included a central 1 mm disc and inner and outer rings of 3 and 6 mm, respectively. The central foveal subfield thickness (i.e., central macular thickness) bounded by the innermost 1 mm diameter circle was calculated. The overall average macular thickness (cube average thickness) and overall macular cube volume over the entire grid area were also obtained from the computational software output based on the proportional contribution of the regional macular thicknesses.

The Ganglion Cell Analysis algorithm processes data from 3-dimensional volume scans using the macular 512 x 128 or 200 x 200 acquisition protocol. The algorithm identifies the outer boundary of the RNFL and the outer boundary of the IPL. The difference between the RNFL and the IPL outer boundary segmentations yields the combined thickness of the RGC layer and the IPL. It provides a measurement of the macular GCIPL thickness within a 14.13-mm^2^ elliptical annulus area centered on the fovea. The mean, minimum, and 6 individual sector (superior, superonasal, inferonasal, inferior, inferotemporal, and superotemporal) GCIPL thicknesses were provided. The minimum GCIPL measurement is determined by sampling 360 measurement spokes extending from the center of the fovea to the edge of the ellipse in 1-degree intervals and selecting the spoke with the lowest average.[24]

All obtained images had signal strengths of ≥ 6. All scans were required to specifically center the optic disc or the fovea. Images with inaccuracies caused by segmentation algorithm errors, involuntary saccades or blinking artifacts were excluded from the analysis.

### Statistical analyses

The composite memory score was estimated by averaging the z scores of the immediate recall, delayed recall, and recognition tests of SVLT and ROCF.[20] The composite executive function score was estimated by averaging the z scores of the animal fluency, COWAT, and color reading score of the Stroop test. The composite attention score was estimated by averaging the z scores from the forward and backward digit span tests. These z scores were based on the means and standard deviations of each measurement in the age- and education-matched control group.[25] A z score is defined as the point at which a score falls within the distribution of scores for normal subjects; a z score of +2.0 corresponds to a score that is 2 standard deviations (SDs) above the mean score.

Demographic and clinical characteristics were compared among the AD, MCI, and control groups using analysis of variance (ANOVA) for continuous variables and the Chi-square test for categorical variables. Neuropsychological test scores, MMSE, and the CDR-Sum of Boxes (CDR-SB) were compared between the MCI and AD groups using an independent t-test. The global CDR score was compared between the MCI and AD groups using Fisher’s exact test.

We selected one eye from each participant randomly for the final analysis. ANOVA tests with post hoc analyses based on the Scheffe method were performed to characterize the differences in OCT parameters among CDR groups. The relationship of the CDR-SB, MMSE, and neuropsychological test scores with OCT parameters were evaluated using a linear regression model adjusted according to age and sex for patients with MCI or AD. We used the Mann-Whitney U test to examine differences in the OCT measurements at baseline between the patients who converted to AD dementia (MCI_AD_) and those who were still classified as MCI (MCI_MCI_) at the time of follow-up examinations. Generalized estimating equation (GEE) models were used to evaluate the associations between baseline OCT parameters and changes in MMSE and CDR-SB scores at 2 years after the baseline values were obtained. All statistical analyses were performed using SPSS 19.0 (SPSS, Chicago, IL, USA). *p* values less than 0.05 were considered statistically significant.

## Results

The baseline characteristics of the AD, MCI, and control groups are summarized in Table 1. No significant differences were observed among the three groups in terms of age, diabetes mellitus, spherical equivalent, and mean intraocular pressure. The proportion of female subjects was higher in the AD group than in the MCI and control groups (*p* = 0.001). There was no difference in the proportion of APOE ε4 carriers between the AD and MCI groups. The patients with AD had higher CDR-SB scores and lower scores on the MMSE and each cognitive domain except for the composite executive function score compared with MCI patients. Thirty-three (78.6%) of the 42 patients with AD and 21 (80.8%) of the 26 patients with MCI were re-evaluated after 2 years. Fourteen subjects were lost to follow-up. All subjects were examined by the same neuropsychologist. There were no significant differences in age, gender, education, and CDR-SB score at baseline between the subjects who discontinued the study and those who completed it.

**Table 1:** Baseline demographic and clinical characteristics of the participants.

	AD (n = 42)	MCI (n = 26)	Control (n = 66)	*P*
Age, years	76.8 ± 8.7	74.7 ± 7.8	73.8 ± 7.5	0.16
Female	38 (90.5%)	16 (61.5%)	38 (57.6%)	**0.001**
Diabetes mellitus	5 (11.9%)	2 (7.7%)	9 (13.6%)	0.73
Intraocular pressure, mmHg	13.40 ± 2.61	13.50 ± 2.30	13.78 ± 2.86	0.75
Spherical equivalent, diopters	-0.02 ± 1.62	0.10 ± 2.01	0.30 ± 1.19	0.55
Carriers with APOE ε4	16/30 (53.3%)	11/22 (50.0%)		0.81
Composite memory score	-1.88 ±1.11	-1.20 ± 1.20		**0.049**
ROCF copy, z score	-2.52 ± 3.65	-0.67 ± 2.61		**0.04**
Short form of K-BNT, z score	-2.11 ± 2.36	-0.77 ± 2.07		**0.02**
Composite attention score	-1.10 ± 1.18	-0.28 ± 1.05		**0.006**
Composite executive function score	-1.66 ± 1.27	-1.04 ± 0.70		0.08
MMSE	14.1 ± 5.5	23.1 ± 4.6		**<0.001**
CDR-SB	7.06 ± 4.24	1.67 ± 1.02		**<0.001**
CDR 0.5: CDR 1: CDR 2: CDR 3	12: 19: 8: 3	26: 0: 0: 0		**<0.001**

Values are given as mean ± standard deviation or number (%). AD = Alzheimer’s disease; MCI = mild cognitive impairment; APOE = apolipoprotein E; ROCF = Rey Osterrieth Complex figure; K-BNT = Korean version of the Boston Naming Test; MMSE = Mini Mental State Examination; CDR = Clinical Dementia Rating scale; CDR-SB = CDR-Sum of Boxes.

Comparisons of OCT measurements among the CDR groups are denoted in Table 2. The average, superior, and nasal RNFL thicknesses were significantly different among the CDR groups. However, there were no significant differences between each of the CDR groups in a post-hoc analysis except superior RNFL difference between CDR 0.5 and CDR 0 groups. The central macular thickness, macular cube volume, and average macular cube thickness were significantly different among the CDR groups. The central macular thickness was significantly different between the CDR ≥ 2 and CDR 0.5 groups. The macular cube volume was significantly different between the CDR ≥ 2 and CDR 0 groups as well as between the CDR 1 and CDR 0 groups. The macular cube average thickness was significantly different between the CDR ≥ 2 and CDR 0 groups. The average, minimum, and six sectoral GCIPL thicknesses were significantly different among the CDR groups. In our post-hoc analysis, we found that the average and minimum GCIPL thicknesses as well as the GCIPL thicknesses of the superotemporal, superonasal, inferonasal, inferior, and inferotemporal sectors in the patients with CDR ≥ 2 were significantly reduced compared with the CDR 0 controls. Furthermore, the average and inferior GCIPL thicknesses in patients with CDR 1 were significantly reduced compared with CDR 0 controls.

**Table 2:** Retinal nerve fiber layer (RNFL) and ganglion cell inner plexiform layer (GCIPL) thicknesses according to the clinical dementia rating scale (CDR).

	CDR 0 (n = 66)	CDR 0.5 (n = 38)	CDR 1 (n = 19)	CDR ≥ 2 (n = 11)	*P*^‡^
RNFL thickness					
Average, μm	93.47 ± 9.91	88.73 ± 9.25	90.26 ± 9.71	86.70 ± 9.20	**0.04**
Temporal, μm	65.44 ± 11.23	67.11 ± 11.11	70.21 ± 11.27	63.30 ± 10.62	0.31
Superior, μm	116.53 ± 14.61	107.57 ± 15.80*	107.05 ± 15.96	104.20 ± 14.28	**0.004**
Nasal, μm	70.94 ± 9.10	68.86 ± 7.54	67.00 ± 8.96	62.60 ± 10.06	**0.03**
Inferior, μm	120.45 ± 16.63	111.65 ± 18.00	115.37 ± 17.82	114.20 ± 16.95	0.09
GCIPL thickness					
Average, μm	79.11 ± 6.49	77.57 ± 6.89	73.68 ± 9.55*	70.45 ± 8.53*	**0.001**
Minimal, μm	73.70 ± 10.39	71.11 ± 11.70	65.26 ± 17.18	57.55 ± 17.28*^†^	**<0.001**
Superotemporal, μm	78.92 ± 6.67	78.20 ± 6.45	73.37 ± 12.46	71.55 ± 8.41*	**0.004**
Superior, μm	80.20 ± 7.69	79.69 ± 8.13	75.26 ± 12.17	73.82 ± 8.27	**0.03**
Superonasal, μm	81.11 ± 8.37	80.11 ± 9.96	75.38 ± 12.47	70.64 ± 10.90*	**0.003**
Inferonasal, μm	78.74 ± 8.55	77.66 ± 7.99	73.68 ± 8.33	70.82 ± 10.15*	**0.01**
Inferior, μm	76.74 ± 6.97	73.51 ± 9.47	69.58 ± 11.35*	64.91 ± 14.35*	**<0.001**
Inferotemporal, μm	79.09 ± 7.36	76.63 ± 8.51	74.79 ± 6.39	71.18 ± 10.83*	**0.009**
Central macular thickness, μm	242.88 ± 20.92	244.68 ± 22.89	239.58 ±20.84	223.27 ± 24.41^†^	**0.03**
Macular cube volume, mm^2^	9.82 ± 0.44	9.65 ± 0.52	9.45 ± 0.58*	9.35 ± 0.38*	**0.002**
Macular cube average thickness, μm	274.76 ± 12.37	270.21 ± 14.49	264.95 ± 16.25	261.27 ± 10.73*	**0.003**

*p < 0.05 vs. CDR 0 group;

^†^*p* <0.05 vs. CDR 0.5 group;

^‡^*p* values by analysis of variance.

The relationship between baseline cognitive function scores and OCT parameters among patients with AD or MCI are presented in Table 3. The CDR-SB score exhibited significant negative relationships with the average GCIPL thickness and GCIPL thickness in the superotemporal, superonasal, and inferonasal sectors. In addition, the composite memory score exhibited significant positive associations with the average GCIPL thickness and the GCIPL thickness in the superotemporal, inferonasal, and inferotemporal sectors. The central macular thickness exhibited a significant positive association with the MMSE score.

**Table 3:** Relationship of the baseline cognitive function scores with the thicknesses of retinal nerve fiber layer (RNFL) and ganglion cell inner plexiform layer (GCIPL) in the patients with Alzheimer’s disease or mild cognitive impairment.

	MMSE	CDR-SB	Composite memory score	ROCF copy	Short form of K-BNT	Composite Attention score	Composite executive function score
RNFL thickness							
Average	-0.057 (0.086)	-0.047 (0.056)	0.008 (0.019)	0.002 (0.046)	0.003 (0.031)	-0.024 (0.016)	-0.013 (0.019)
Temporal	-0.029 (0.072)	-0.017 (0.047)	0.018 (0.015)	0.011 (0.038)	-0.025 (0.026)	-0.012 (0.014)	0.006 (0.014)
Superior	-0.018 (0.052)	-0.023 (0.034)	-0.007 (0.012)	-0.024 (0.028)	0.013 (0.019)	-0.004 (0.010)	-0.015 (0.011)
Nasal	0.118 (0.093)	-0.114 (0.059)	0.025 (0.023)	0.053 (0.055)	0.033 (0.036)	-0.015 (0.020)	0.006 (0.021)
Inferior	-0.053 (0.045)	-0.003 (0.029)	0.001 (0.010)	0.005 (0.025)	-0.006 (0.016)	-0.015 (0.008)	-0.006 (0.010)
GCIPL thickness							
Average	0.153 (0.103)	-0.150* (0.066)	0.060* (0.023)	0.009 (0.056)	0.042 (0.036)	0.009 (0.019)	-0.009 (0.027)
Minimum	0.050 (0.056)	-0.060 (0.036)	0.010 (0.013)	-0.021 (0.031)	0.020 (0.021)	0.003 (0.011)	-0.018 (0.012)
Superotemporal	0.163 (0.091)	-0.121* (0.059)	0.066* (0.024)	0.018 (0.048)	0.036 (0.032)	0.007 (0.017)	0.013 (0.032)
Superior	0.109 (0.088)	-0.089 (0.057)	0.041 (0.022)	0.012 (0.046)	0.032 (0.031)	0.016 (0.016)	0.001 (0.025)
Superonasal	0.144 (0.076)	-0.109* (0.049)	0.029 (0.017)	0.039 (0.042)	0.049 (0.027)	0.018 (0.014)	0.005 (0.017)
Inferonasal	0.106 (0.099)	-0.149* (0.063)	0.045* (0.020)	-0.014 (0.055)	0.054 (0.035)	0.011 (0.019)	-0.007 (0.022)
Inferior	0.049 (0.075)	-0.081 (0.048)	0.024 (0.017)	-0.047 (0.043)	0.014 (0.028)	-0.003 (0.015)	-0.030 (0.018)
Inferotemporal	0.092 (0.101)	-0.115 (0.065)	0.062* (0.019)	0.052 (0.058)	-0.001 (0.036)	-0.005 (0.019)	-0.001 (0.022)
Central macular thickness	0.096* (0.034)	-0.038 (0.023	0.002 (0.010)	0.009 (0.023)	0.014 (0.014)	0.014* (0.007)	0.005 (0.010)
Macular cube volume	0.123 (1.572)	-1.613 (0.999)	0.281 (0.305)	-0.186 (0.791)	0.196 (0.549)	-0.115 (0.290)	-0.314 (0.301)
Macular cube average thickness	0.010 (0.056)	-0.061 (0.036)	0.010 (0.011)	-0.006 (0.028)	0.008 (0.020)	-0.003 (0.010)	-0.010 (0.011)

Values are given as beta (standard error) by linear regression model adjusted with age and sex. MMSE = Mini Mental State Examination; CDR-SB = Clinical Dementia Rating scale-Sum of Boxes; ROCF = Rey Osterrieth Complex figure; K-BNT = Korean version of the Boston Naming Test.

*p < 0.05

Comparisons of the RNFL and GCIPL thicknesses at baseline between the MCI_AD_ and MCI_MCI_ groups are summarized in Table 4. Nine patients in the MCI_AD_ group progressed to dementia of the Alzheimer’s type. The temporal RNFL thickness at baseline was significantly thinner in the MCI_AD_ group compared to the MCI_MCI_ group (*p* = 0.04). The average and minimum GCIPL thicknesses as well as the GCIPL thickness in the inferonasal, inferior, and inferotemporal sectors at baseline were significantly thinner in the MCI_AD_ group compared to the MCI_MCI_ group.

**Table 4:** Comparisons of the baseline thicknesses of retinal nerve fiber layer (RNFL) and ganglion cell inner plexiform layer (GCIPL) between patients with mild cognitive impairment who converted to Alzheimer’s disease (MCI_AD_) and stable MCI patients (MCI_MCI_).

	MCI_AD_ (n = 9)	MCI_MCI_ (n = 12)	*P*
RNFL thickness			
Average, μm	86.56 ± 10.20	92.58± 9.78	0.19
Temporal, μm	60.78 ± 11.05	71.33 ± 10.32	**0.04**
Superior, μm	112.22 ± 18.08	111.75 ± 16.49	0.92
Nasal, μm	69.00 ± 6.91	70.08 ± 7.48	0.70
Inferior, μm	103.89 ± 17.22	117.58 ± 17.74	0.11
GCIPL thickness			
Average, μm	73.56 ± 6.48	81.90 ± 5.47	**0.01**
Minimum, μm	65.33 ± 11.02	79.00 ± 5.54	**0.008**
Superotemporal, μm	76.56 ± 5.68	81.10 ± 7.39	0.24
Superior, μm	75.33 ± 9.08	82.80 ± 5.79	0.21
Superonasal, μm	76.56 ± 9.41	83.20 ± 4.94	0.13
Inferonasal, μm	72.78 ± 6.82	81.50 ±6.12	**0.02**
Inferior, μm	68.67 ± 7.70	80.20 ±6.29	**0.004**
Inferotemporal, μm	71.22 ± 10.78	82.60 ± 5.36	**0.006**
Central macular thickness, μm	257.22 ± 9.11	251.00± 25.46	0.51
Macular cube volume, mm^2^	9.64 ± 0.36	9.92 ± 0.49	0.19
Macular cube average thickness, μm	270.33 ± 10.49	278.08 ± 13.64	0.17

Values are given as mean ± standard deviation.

The change in CDR-SB from baseline to 2 years had significant negative associations with the macular cube volume, macular cube average thickness, average and minimum GCIPL thicknesses, and GCIPL thicknesses of the superotemporal, superior, superonasal, and inferonasal sectors at baseline (Table 5). The change in MMSE from baseline to 2 years had significant positive associations with central macular thickness and GCIPL thickness in the superonasal sector.

**Table 5:** The impact of the thicknesses of retinal nerve fiber layer (RNFL) and ganglion cell inner plexiform layer (GCIPL) at baseline on the changes of MMSE and CDR-SB at 2 years in the patients with mild cognitive impairment or Alzheimer’s disease.

	MMSE		CDR-SB	
	Beta (SE)	*P*	Beta (SE)	*P*
RNFL thickness				
Average	-0.055 (0.070)	0.43	-0.013 (0.049)	0.79
Temporal	-0.050 (0.063)	0.43	0.021 (0.046)	0.66
Superior	-0.015 (0.045)	0.73	-0.014 (0.027)	0.61
Nasal	0.073 (0.070)	0.30	-0.049 (0.051)	0.33
Inferior	-0.036 (0.038)	0.34	-0.002 (0.024)	0.92
GCIPL thickness				
Average	0.139 (0.083)	0.09	-0.150 (0.054)	**0.006**
Minimum	0.056 (0.054)	0.30	-0.069 (0.032)	**0.03**
Superotemporal	0.135 (0.071)	0.06	-0.128 (0.055)	**0.02**
Superior	0.096 (0.057)	0.09	-0.102 (0.043)	**0.02**
Superonasal	0.141 (0.061)	**0.02**	-0.121 (0.038)	**0.001**
Inferonasal	0.103 (0.091)	0.26	-0.130 (0.051)	**0.01**
Inferior	0.047 (0.081)	0.56	-0.077 (0.046)	0.09
Inferotemporal	0.091 (0.106)	0.39	-0.110 (0.068)	0.11
Central macular thickness	0.083 (0.026)	**0.001**	-0.029 (0.020)	0.13
Macular cube volume	0.542 (1.321)	0.68	-1.700 (0.728)	**0.02**
Macular cube average thickness	0.024 (0.048)	0.62	-0.063 (0.026)	**0.02**

Values are given as beta (standard error) by generalized estimating equation. MMSE = Mini Mental State Examination; CDR-SB = Clinical Dementia Rating scale-Sum of Boxes.

## Discussion

In this study, we present data indicating that macular GCIPL and peripapillary RNFL reduction, as assessed by HD-OCT, could discriminate dementia stages as measured by CDR. The reduction in GCIPL was also associated with memory decline and incremental changes in CDR-SB score in patients with MCI or AD. Thus, a GCIPL reduction at baseline was predictive of the progression of AD and MCI over a 2-year follow-up period.

Advances in ocular imaging techniques, particularly advances in OCT, have enabled visualization of individual retinal layers and quantitative assessment of each layer. Imaging studies have reported that macular changes reflect neurodegenerative changes in AD. Marziani et al. reported significant reductions in combined RNFL and GCL thickness in macular region assessed by SD-OCTs (RTVue-100, Optovue and Spectralis OCT, Heidelberg) in 21 AD patients compared with healthy controls.[12] Garcia-Martin et al. measured macular thickness using 3D OCT-100 (Topcon, Japan) and proposed that the first affected area of the retina in mild AD may be the macular area.[13] Cheung et al. measured the GCIPL without including the RNFL using the SD-OCT (Cirrus OCT, Carl Zeiss) ganglion cell analysis algorithm.[17] [14] Gao et al. reported reduced macular cube volume obtained by Cirrus SD-OCT in MCI and AD patients.[15]

The average RNFL thickness and that of the superior and nasal sectors differed significantly among the CDR groups included in this study. However, there were no significant differences between each CDR group in a post-hoc analysis except superior RNFL difference between CDR 0.5 and CDR 0 groups. On the other hand, the average and minimum GCIPL thicknesses as well as the GCIPL thickness of all six sectors were significantly different among the CDR groups. These findings suggest that GCIPL thickness could discriminate between CDR stages more sensitively than RNFL thickness. Our study also suggests that macular cube volume and average thickness as well as central macular thickness could discriminate between CDR stages.

The reduction of central macular thickness was associated with a reduction in MMSE and composite attention scores. Our results are consistent with previous imaging studies reporting a reduction in total macular volume obtained by TD-OCT (OCT Model 3000, Carl Zeiss) in patients with AD, which is correlated with MMSE score,[26] and reduction in macular ganglion cell complex (GCC) thickness which was related to MMSE score in AD patients.[14]

When we further examined correlation between average GCIPL thickness and each of specific domains of neuropsychiatric ability, composite memory score was clearly correlated with GCIPL thickness. Visual learning, verbal ability, attention ability, and executive function domains were not directly related to macular average GCIPL thickness. A large number of studies have examined the accuracy of cognitive tests to predict progression from MCI to AD, and studies have revealed that a range of episodic memory tests are excellent predictors of future decline.[27] AD is a progressive degenerative disorder of insidious onset, characterized, in its earliest stages, by declarative memory loss. Research diagnostic criteria for AD proposed by an expert consensus group led by Dubois set early and substantial impairment in episodic memory as core diagnostic criteria for AD.[28] Significant correlation between inner retinal thinning and memory domain score may be a result of an episodic memory deficit as the predominant initial complaint in most cases of AD. To our knowledge, no studies have assessed specifically relationships between memory function impairment and retinal inner layer thickness measurements in the AD patients.

In this study, reduced average and minimum GCIPL thicknesses and lower GCIPL thicknesses in the inferonasal, inferior, and inferotemporal sectors at baseline were associated with the conversion to AD dementia from MCI. The lower temporal quadrant RNFL thickness at baseline was also related with the conversion to AD dementia from MCI. Thinner temporal quadrant RNFL corresponds to the thinner papillomacular bundle which trajects to macula. These findings suggest that macular GCIPL parameters seem to be more predictive of the conversion to AD dementia from MCI compared to RNFL parameters.

We found for the first time that baseline macular OCT parameter values predicted subsequent cognitive decline. The increment of CDR-SB in patients with MCI or AD after 2 years follow-up was associated with lower average and minimum GCIPL thicknesses as well as lower GCIPL thicknesses in the superotemporal, superior, superonasal, and inferonasal sectors at baseline. The increment of CDR-SB in these patients was also related to reductions in macula cube volume and average thickness. Reduced central macular and superonasal GCIPL thicknesses were associated with a decline in the MMSE score in patients with MCI or AD at the 2-year follow-up. However, RNFL thickness was not associated with changes in CDR-SB and MMSE in patients with MCI or AD upon the 2-year follow-up. These findings suggest that GCIPL thickness as well as macula cube volume and thickness could predict the disease progression of MCI and AD. There is increasing evidence that in neurodegenerative conditions, morphological alterations of dendrites occur both in the retina and in the central nervous system.[29] Previous histologic studies,[8, 10, 30] animal studies,[31, 32] and imaging studies[33] have supported the hypothesis that changes in RGCs and optic nerve axons reflect the neurodegenerative pathology of AD. The exact biological mechanism for a causal relationship between dementia and changes in macular area remains should be further investigated.

Macular GCIPL is based on a sampling of approximately 50% of the RGC population whose cell bodies are over 10 to 20 times the diameter of their axons.[34] RGC neuronal loss may begin in the macula due to the density of the RGC population in this region.[34] In addition, the macula is the retinal region with the highest activity which implies possibility of neuronal hyperexcitation. Moreover, the fovea does not contain blood vessels which can lead possible undersupply with oxygen and nutrients. Interestingly, in postmortem histological studies, the substantial loss and prominent pathological alteration of RGCs was observed in the macular area in AD patients.[35, 36] A previous animal study reported that amyloid-β deposits in the RGC layer led directly to neurodegeneration in the retina of a transgenic mouse model of AD.[37] In a recent animal study, dendritic changes in RGCs preceded cell loss in a mouse model of AD, suggesting that the inner retinal layer may represent a useful marker for the early detection of neurodegeneration.[38]

Our study has some limitations. The first is its relatively small sample size, which may have reduced the significance of our findings. Second, normal controls were not evaluated by extensive neuropsychological testing. Some elderly individuals exhibiting minor cognitive decline may have therefore been included in the control group, which could weaken the differences observed between the groups. Third, longitudinal OCT measurement data were not obtained, necessitating further study to confirm the association between the changes in OCT measurements and cognitive decline. Fourth, non-AD dementias may also present retinal involvement measured by OCT. Further studies with different types of dementia are required to elucidate usefulness of retinal biomarkers.

The strength of this study lies in the fact that we evaluated the cross-sectional association between the CDR scale and cognitive domain score with retinal thickness in AD and MCI patients. Furthermore, to the best of our knowledge, this study was the first to evaluate the impact of retinal thickness on the progression of AD and MCI with a prospective design. In conclusion, reductions in the macular total thickness and GCIPL thickness are associated with disease severity and cognitive function in MCI and AD and are predictive of the progression these diseases.
